# Evaluation of Testicular Toxicity Following Short-term Exposure to Cypermethrin in Albino Mice

**DOI:** 10.4103/0971-6580.68344

**Published:** 2010

**Authors:** N. Prakash, Kumar M. Vijay, U. Sunilchandra, BH. Pavithra, A. Pawar

**Affiliations:** Department of Veterinary Pharmacology and Toxicology, Veterinary College, Karnataka Veterinary Animal and Fisheries Sciences University, Bidar - 585 401, India; 1 Department of Anatomy and Histology, Veterinary College, Karnataka Veterinary Animal and Fisheries Sciences University, Bidar - 585 401, India

**Keywords:** Albino mice, histopathology, testosterone, testes, ultrastructural changes, α-Cypermethrin

## Abstract

The present study was undertaken to assess the testicular toxicity following short-term exposure to cypermethrin (α-CP) in albino mice. Cypermethrin was dissolved in *arachis oil* and administered to two groups of mice (*n* = 12/group) orally at the dose rate of 250 mg/kg body weight, once a day for 28 days. Fifty percent of the animals in both the groups were sacrificed on day 14 and the remaining on day 28. Plasma samples were subjected to radioimmunoassay to determine testosterone levels. The testes were collected to determine the cholesterol levels and the activity of transaminases (AST and ALT) or epididymal alkaline phosphatase (ALP). Histological study of testicular tissue was also undertaken to examine the α-CP-induced ultrastructural changes using transmission electron microscopy (TEM). α-CP significantly (*P*<0.05) increased the activities of testicular AST (1.36±0.12 vs. 1.19±0.10), ALT(1.78±0.11 vs. 1.36±0.09), and significantly (*P*<0.05) decreased the testosterone levels (0.86±0.24 vs. 1.72±0.18). Testicular cholesterol levels were elevated in treated animals as compared to control (1.81±0.16 vs. 1.42±0.08). Epididymal alkaline phosphatase (ALP) activity was also decreased significantly (*P*<0.05) in treated animals (1.10±0.20 vs. 1.64±0.1). Histological studies on day 28 revealed rupture of spermatogonic cell membrane, shrinkage in the nucleus, stages of apoptosis, condensation of chromatin, and decreased cytoplasmic organelles. The study suggested that short-term exposure to α-CP in albino mice induced toxicopathological lesions in testicular tissue leading to decreased plasma testosterone levels.

## INTRODUCTION

Synthetic pyrethroids represent one quarter of the insecticides used in agriculture all over the world. These are a diverse class of more than thousand powerful, broad-spectrum insecticides used to control insect pests in animals, agriculture, households, and stored products. Although they are based on the chemical structure and biological activity of pyrethrum, an extract from plants in the genus *Chrysanthemum*, the development of synthetic pyrethroids has involved extensive chemical modifications to make compounds more toxic and less rapidly degraded by light. Widespread use of insecticides in animal husbandry and agriculture for many years can lead to their contamination in the food chain and the environment.[1]

While the agricultural utilization of pyrethroids derived from natural pyrethrins is limited due to their low photostability, synthetic pyrethroids of the second and third generations are photostable[2] and highly effective against broad spectrum of insects.[3] Although pyrethroids posses wide mammalian–insect toxicity ratio, they are capable of disrupting endocrine function upon subacute or chronic exposure. α-cypermethrin (α-CP) is a synthetic pyrethroid used not only as ectoparasiticide in animals but also employed as insecticide extensively in agriculture and public health programs. The technical-grade cypermethrin is the racemic mixture of eight isomers (four cis- and four trans- isomers) out of which two stereoisomers are believed to be the most active isomer and called as α-CP.[4] Some of the toxic actions of α-CP have been reported earlier.[4] Cypermethrin is considered as one of the endocrine disruptors.[5] The present study was undertaken to evaluate the testicular toxicity following short-term exposure to cypermethrin in albino mice.

## MATERIALS AND METHODS

Alfa-cypermethrin [cyano-(3-phenoxy-phenyl) methyl 1,3-(2,2-dichloroehtyl)-2,2-dimethylcyclopropane carboxylate] (α-CP>99% pure) was procured from Gharda Chemicals Ltd., Mumbai. Twenty-four healthy Swiss albino mice males weighing between 30 and 40 g were divided into two equal groups consisting of 12 animals each. All mice were kept under laboratory conditions of temperature (24±1° C) and humidity (60±5%). They were given pellet feed (Amruta feeds Ltd., Pune, India) and drinking water *ad libitum*. A twelve-hour day and night cycle was maintained in the animal house. Cypermethrin was dissolved in arachis oil (1:9) and administered to the mice in treated group orally at the dose rate of 250 mg/kg body weight once a day for 28 days and the control group received arachis oil only. Body weights of individual animals were recorded daily. Six animals in each group were sacrificed on day 14 of administration of cypermethrin and remaining 12 animals in both the groups were sacrificed on day 28 of the experiment.

The animals were fasted overnight, sacrificed by decapitation and trunk blood was collected from the heart. Testes were removed, weighed, and frozen immediately in liquid nitrogen and stored at –20°C till further processing. Freshly removed testes and epididymus of animals were trimmed from extraneous material using chilled saline solution and homogenized in 0.25 M ice-cold sucrose solution (10%w/v) in tissue homogenizer. The homogenates were centrifuged (700g) at room temperature for 10 min to remove cell debris. The supernatants of the testes was used for the estimation of aspartate transaminase (AST), alanine transaminase (ALT) levels,[6] cholesterol,[7] and plasma testosterone levels.[8] IFCC[9] method was followed for the estimation of membrane-bound enzyme alkaline phosphotase (ALP) activity in the epididymus.

Histopathological examination was done by formal-Bouin’s fixed tissue, dehydrated in graded alcohols, processed to prepare paraffin-embedded blocks, and routine microtomy was followed for obtaining 5–8-*μ*m thick paraffin sections, which were routinely stained by hematoxylin–eosin (HandE)[10] and observed under light microscope. Representative samples of testes were also fixed in glutaraldehyde fixative and were studied under transmission electron microscopy (TEM). All the values were expressed as mean ± SE. Statistical analysis was done using Student’s *t* test.[11]

## RESULTS AND DISCUSSION

**Figure 1:**
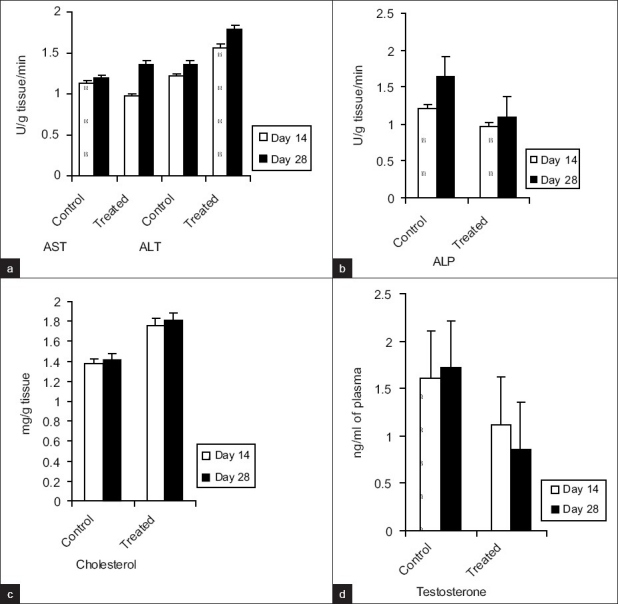
Effect of α-CP on testicular (A) transaminases (AST and ALT) activity, (B) epididymal alkaline phosphatase (ALP), (C) cholesterol, and (D) plasma testosterone levels

Short-term α-CP exposure to male Swiss albino mice did not cause any significant change in the body weights of the treated group compared to control group. However, α-CP exposure to male mice produced an appreciable increase in the testicular wet-weights as noticed on day 14 and day 28 of the experiment compared to the control group. Similarly an increase in the weight of accessory sex organs, *viz*. seminal vesicles, prostate gland, and epididymis, was found (data not included). The effect of α-CP on certain testicular parameters is presented in Table 1. α-CP significantly (*P*<0.05) increased the activities of testicular AST, ALT [Figure 1a], and significantly (*P*<0.05) decreased the epididymal ALP levels [Figure 1b]. However, information regarding biochemical changes associated with α-CP exposure on testicular tissue of experimental animals is scanty.[12] Chemical-induced cellular alteration varies from simple increase in metabolism to death of cell.[13] The increase or decrease in enzyme activity is related to the intensity of cellular damage; therefore, increase in transaminase activity (AST and ALT) along with decrease in the epididymal ALP activity may be the consequences of α-CP-induced cellular alteration in testicular tissues. α-CP significantly (*P*<0.05) increased the cholesterol levels [Figure 1c] and significantly (*P*<0.05) decreased the plasma testosterone levels [Figure 1d] on day 28. Testosterone levels in plasma decreased significantly in the treated group even though there was an increase in the cholesterol levels, which may be due to α-CP effect on the activity of two steroidogenic enzymes, viz. 3β- and 17β -hydroxy steroid dehyrogenase (17β-OHSD) resulting in improper androgen synthesis that is the conversion of ∆^5^ androstenediol to testosterone and disruption of steroidogenesis.[14] Although pyrethroids posses wide mammalian–insect toxicity ratio, they are capable of disrupting endocrine function upon subacute or chronic exposure.[15]

**Table 1:** Effect of α -cypermethrin (α -CP) on biochemical parameters of testes after administration (250 mg/kg, p.o.) in albino mice

	Group I (Control)		Group II (α-CP treated)	
	Day 14	Day 28	Day 14	Day 28
AST (U/ml)	1.13±0.09	1.19±0.10	0.97±0.08*	1.36±0.12**
ALT (U/ml)	21±0.09	1.36±0.09	1.56±0.15*	1.78±0.11**
Cholesterol	1.37±0.21	1.42±0.08	1.76±0.23*	1.81±0.16**
ALP (U/ml)	1.21±0.11	1.64±0.1	0.97±0.08*	1.10±0.20**

Values are mean ± SE;

* *P*<0.05; Note: * and

** denote significantly different *P*<0.05 on day 14 and day 28, respectively

**Figure 2:**
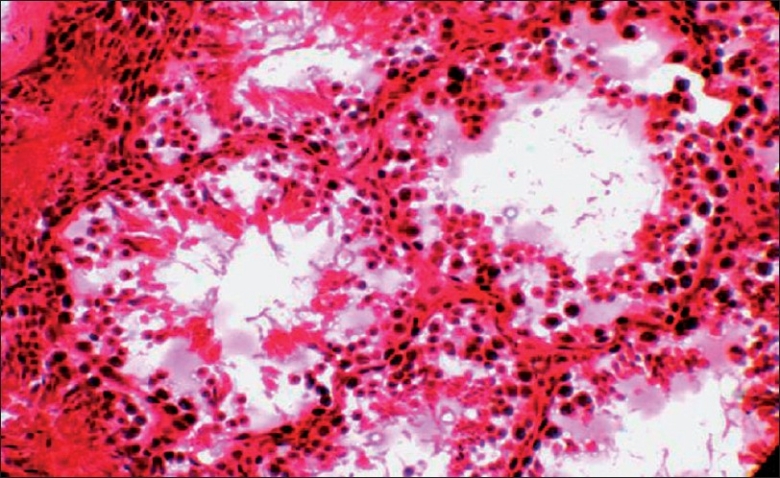
Transverse section of testes showing different spermatogonic cells in the semineferous tubules in control group (HandE, ×10) on day 28

**Figure 3:**
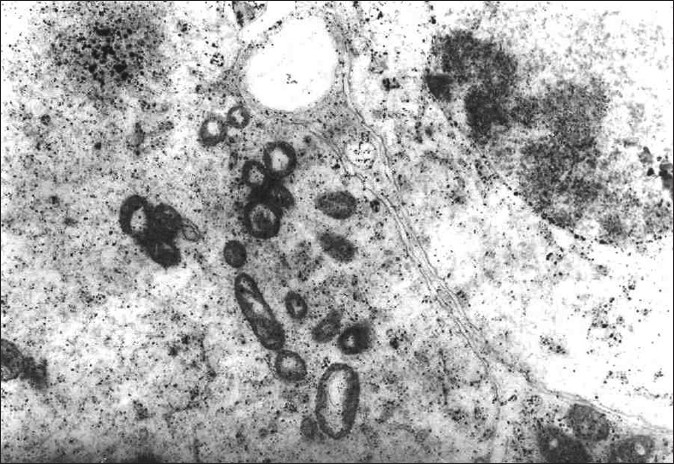
Transmission electron microscopy of semineferous tubules showing (a) spermatogonic cells with rupture of cell membrane, (b) increased lipochrome pigment, (c) shrinkage in the nucleus on day 28 (TEM:1.0K × negative)

**Figure 4:**
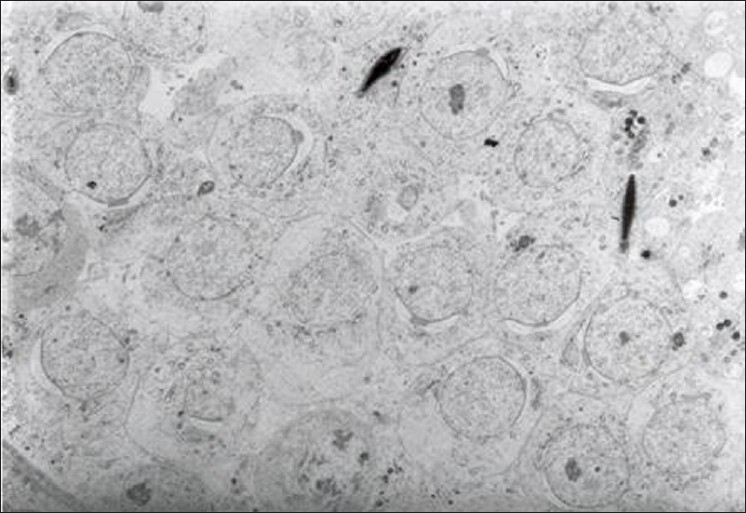
Transmission electron microscopy of testes showing spermatogonic cells lying over basement membrane of semineferous tubule. (a) Early stages of apoptosis; (b) condensation of chromatin; and (c) decreased cytoplasmic organelles on day 28 (TEM: 2.5 K × negative)

Histological examination of testis of control group revealed normal spermatogonic cells in the semineferous tubules [Figure 2]. The section of testis of treated group studied under TEM revealedchanges in spermagonic cells like rupture of cell membrane, shrinkage in the nucleus, stages of apoptosis, condensation of chromatin [Figure 3], and decreased or absence of cytoplasmic organelles [Figure 4]. The histological studies also revealed the pathological damage induced by the α-CP on testicular tissue, which ranges from cell membrane damage of the seminiferous tubules to shrinkage in the nucleus, stages of apoptosis, condensation of chromatin, and decreased cytoplasmic organelles, which was in agreement with Manna *et al*.[12] Due to these biochemical and pathological lesions, testes weight in α-CP-treated group increased significantly compared to control group.

The study indicated the potential toxicity of α-CP on testes, leading to enzymatic alterations in testes as well as disruption of testosterone synthesis.
